# Identification of an aptamer through whole cell-SELEX for targeting high metastatic liver cancers

**DOI:** 10.18632/oncotarget.6988

**Published:** 2016-01-23

**Authors:** Yuan Rong, Hao Chen, Xue-Feng Zhou, Chang-Qing Yin, Bi-Cheng Wang, Chun-Wei Peng, Shao-Ping Liu, Fu-Bing Wang

**Affiliations:** ^1^ Department of Laboratory Medicine & Center for Gene Diagnosis, Zhongnan Hospital of Wuhan University, Wuchang, Wuhan 430071, P.R. China; ^2^ Department of Thoracic and Cardiovascular Surgery, Zhongnan Hospital of Wuhan University, Wuchang, Wuhan 430071, P.R. China; ^3^ Department of Pathology, Zhongnan Hospital of Wuhan University, Wuchang, Wuhan 430071, P.R. China; ^4^ Department of Oncology, Zhongnan Hospital of Wuhan University, Wuchang, Wuhan 430071, P.R. China; ^5^ Hubei Key Laboratory of Tumor Biological Behaviors & Hubei Cancer Clinical Study Center, Wuchang, Wuhan 430071, P.R. China

**Keywords:** hepatocellular carcinoma, migration, invasion, cell-SELEX, aptamer

## Abstract

Hepatocellular carcinoma (HCC) is one of the most deadly human cancers due to its ability of invasion and metastasis. Thus, the approaches to identify potential compounds that inhibit invasion and metastasis of HCC are critical for treatment of this disease. In the present study, we used HCCLM9 cells with high metastatic potential and MHCC97L with low metastatic potential as a model system to study the molecular mechanisms of HCC metastasis. By applying cell- Systematic Evolution of Ligands by Exponential enrichment (SELEX) against living cells, we used HCCLM9 as target cells and MHCC97L cells as control to screen a group of HCC metastasis- and cell-specific DNA aptamers. One of selected aptamers, LY-1, could specifically bind to metastatic HCC with a dissociation constant (Kd) in nanomolar range. *In vitro* studies demonstrated that LY-1 can recognize and bind to membrane protein of metastatic HCC cells. Furthermore, QD605 labeled LY-1 aptamer could recognize HCC cells in both local liver cancer tissues and pulmonary metastatic sites in a xenograft model of HCC with pulmonary metastasis. Further biochemical and immunostaining studies showed that LY-1 could selectively bind to a subpopulation of more metastatic cells in HCCLM9 cells, which express more CK19 and vimentin. Finally, treatment of highly metastatic cells with LY-1 led to reduced migration and invasiveness of HCCLM9 cells *in vitro* and suppression of xenograft growth *in vivo*. Taken together, the present study demonstrated the tumor targeting and tumor suppressive effects of LY-1, which could be a promising molecular probe for metastatic HCC and a potential candidate of chemotherapy for metastatic HCC.

## INTRODUCTION

The prognosis of patients with hepatocellular carcinoma (HCC) is poor due to the invasive and metastatic signatures of this deadly tumor [1, 2]. The metastasis and recurrence of HCC are closely associated with the migration and invasion of the tumor cells, which has been shown to be dependent on the expression of specific surface markers on the metastatic cells [3]. As the cell membrane surface molecules play important roles in cancer pathogenesis, identification of these tumor- and metastasis-related surface markers becomes critical for diagnosis and treatment of HCC [4, 5].

However, the molecular identities of the surface markers for HCC cells are complex which make them extremely difficult to identify through current routine biochemical and molecular biology techniques. Therefore, a method has been developed to enable a quick and high throughput screening of nucleic acid-based ligands that can specifically target the cells of interest with high affinity [6]. This method is called Systematic Evolution of Ligands by Exponential Enrichment (SELEX) [7]. The technology takes repetitive *in vitro* selection and PCR amplification, to enrich nucleic acid-based ligands (aptamers) that are short single-stranded nucleic acid oligomers with a specific three-dimensional configuration, which enables them specifically bind to target molecules on the plasma membrane of their target cancer cells. Previous studies have shown that aptamers function as antibodies in molecular recognition, in addition they have following attractive features: low molecular weight, easy to reproduce, high binding affinity and molecular specificity, easy to modify, fast tissue penetration, and low toxicity to normal tissues [8]. These advantages have made aptamers an excellent alternative as molecular probes in multiple applications such as, bioanalysis, biomedicine, and biotechnology [9]. Aptamers with the three-dimensional structures can bind specifically to their targets, ranging from small molecules to proteins and even whole cells [9, 10]. Several aptamers have been identified against cancer-related proteins, such as EGFR, VEGF, HER3, NF-κB, tenascin-C or prostate-specific membrane antigen (PSMA) [11–16]. Then, the protocol of aptamer selection against whole cancer cells was subsequently developed [6]. Compared with protein-based SELEX, the cell-SELEX can be carried out without prior knowledge of identity of the targets on cell surface [7, 17]. Thus, cell-SELEX is suitable for screening of molecular probes that specifically bound to the surface of tumor cells with complex molecular components [5, 10].

To generate aptamers against the whole living cells, the most important factor to consider is the choice of target and control cells. In general, two cell population derived from same genetic background is required for successful screening of highly specific aptamers from minimal number of SELEX rounds [18]. In the present study, human HCC cell lines MHCC97L with low metastatic potential and HCCLM9 with high metastatic potential were chose for the purpose of screening metastasis specific aptamers [19]. HCCLM9 was originally screened gradually from nude mice bearing MHCC97L cells (detailed cell generation protocol was summarized in Supplementary Figure S1). As a result, the different metastatic potential between HCCLM9 and MHCC97L provides clues and molecular basis for clinical prediction of metastasis and recurrence, and potential targets for interventional therapy for treatment of highly metastatic HCC.

The purpose of our study is to identify aptamers that could serve as a potential marker for metastatic HCC. We applied subtractive cell-SELEX method for generation of aptamers, by using HCCLM9 as the target cells and MHCC97L as a control, based on the fact that these two cell lines have the same genetic background but significant difference in metastasis potential. The enrichment of aptamers pool and the binding affinity and specificity were determined by flow cytometry and immunostaining assays. We found that one of identified aptamers, named LY-1, can specifically recognize protein targets on the surface of metastatic cells. Furthermore, xenograft with lung metastasis mouse model experiments provided in vivo evidence to show the lung metastatic tumor cells can be targeted by LY-1, indicating its potential for treatment of metastatic HCC. More importantly, aptamer LY-1 treatment could inhibit HCC cells invasion and migration *in vitro* and suppress the tumor growth in vivo. Taken together, our results demonstrated that LY-1 could be a potential diagnostic tool and a chemotherapy for metastatic HCC.

## RESULTS

### Selection and enrichment of aptamers specific to metastatic HCC cells

**Figure 1:**
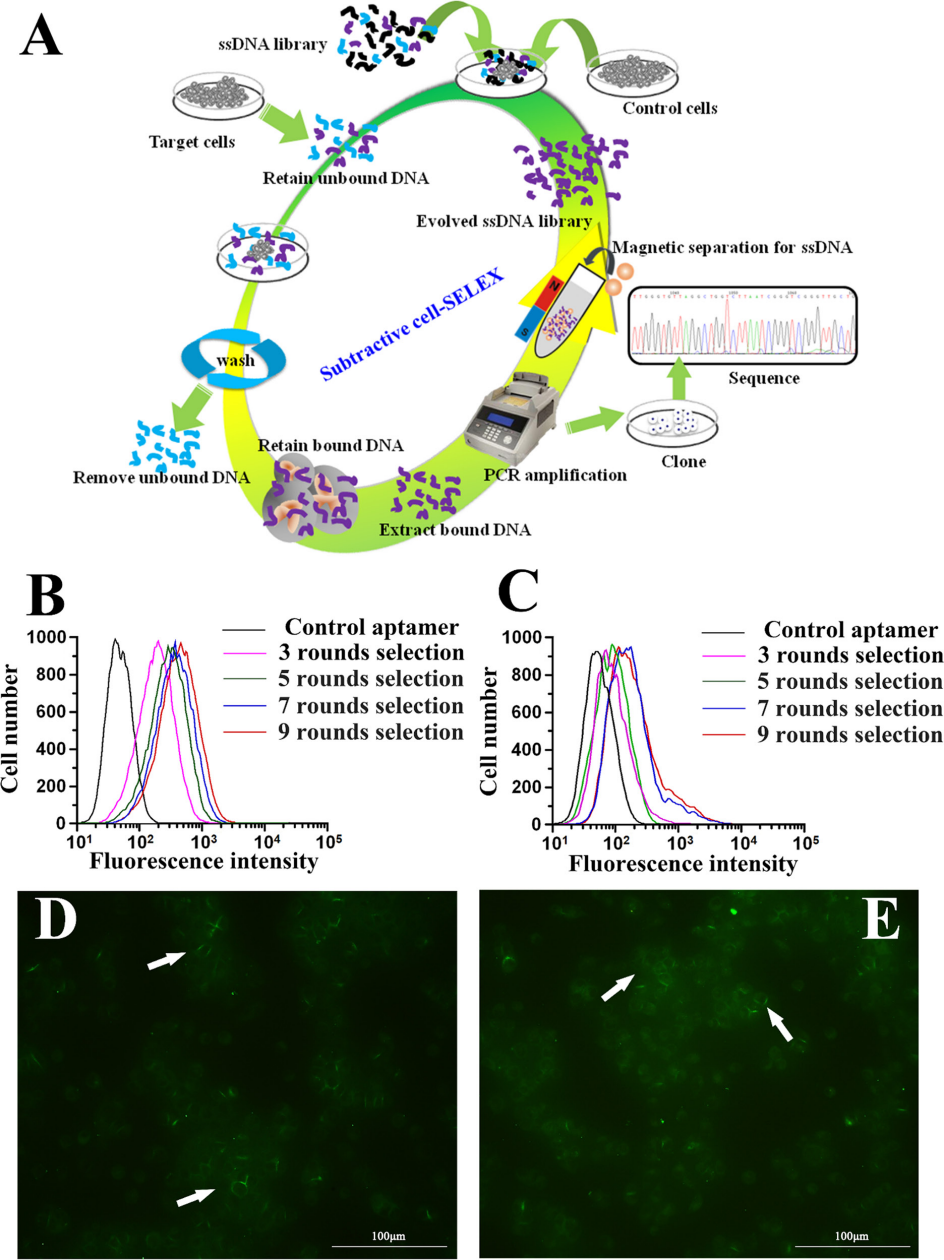
Identification of aptamer that specifically targets metastatic HCC **A.** Schematics of the cell-based aptamer selection in our study. **B, C.** Flow cytometry assay to monitor the binding of selected library with HCCLM9 (target cells) and MHCC97L (subtractive cells). **B.** Increasing fluorescence intensity bound to HCCLM9 with the 3, 5, 7 and 9 rounds of selected library; **C.** Fluorescence intensity bound on MHCC97L with the 3, 5, 7 and 9 rounds selected library by flow cytomtry. With increasing rounds of enrichment, significant increases in fluorescence intensity were detected on HCCLM9 cells (panel B) but not on MHCC97L (panel C), suggesting the enrichment of HCCLM9 specific aptamers. **D.** The fluorescence imaging of the 9 rounds selected library bound to HCCLM9 cells by fluorescent microcopy analysis. **E.** The fluorescence imaging of the 5 rounds selected library bound to HCCLM9 cells by fluorescent microcopy analysis.

The whole cell-SELEX strategy used in our work was illustrated in Figure 1A, with the detailed procedures described in the Materials and Methods section. HCCLM9 cells were used as target (the positive cell line) and MHCC97L for subtractive selection (the negative control cell line). A single-stranded DNA library containing a 40bp central random sequence flanked by two 18bp PCR primers was used in this selection. After denaturation and being placed on ice, the library was incubated with the target cells to allow random DNA sequences to bind to cell membrane. Then the cells were rinsed and removed from supernatant containing unbound DNA sequences, after which the DNA sequences bound to the cell surface were eluted by heating. The collected DNA sequences were amplified by PCR for the next round of selection. From the fourth round of selection, the selected DNA pool could be used to carry out subtractive selection (the selected DNA pool firstly incubated with subtractive cells, then the supernatant containing unbound ssDNA sequence incubated with target cells) to filter out sequences that may bind to the molecules existing on the surface of the subtractive cells. After multi-round selection, the aptamer candidates bound to the target cells were enriched. The enrichment of the DNA pool through successive selection was monitored by flow cytometry (Figure 1B, 1C) and immunofluorescence imaging (Figure 1D, 1E). The increased fluorescence intensity was an indication of the enrichment of cell-binding DNA sequences. With the increasing cycles of selection, there was a steady increase in the fluorescence intensity on the target cells HCCLM9, while there was no significant change of fluorescence intensity on the control cells MHCC97L. Therefore, it was evident that a panel of aptamer probes eventually evolved to have great specificity and high affinity for metastatic HCC cells along with the progression of SELEX cycles.

### Identification and characterization of selected aptamers for metastatic HCC cells

After 9 rounds of selection, the enriched pool presented a considerable increase in affinity for the target cells (Figure 1B, 1D, 1E). On further enrichment (10 and 11 rounds of selection), there was no significant enhancement of the fluorescence intensity on the target cells HCCLM9 (data not shown). Therefore, we decided to work with the pool after 9 rounds of selection. The highly enriched DNA pool was cloned. Fifty clones were subjected to sequence study, but positive results were available from 38 clones, including 23 clones yield a same sequence named as LY-1, 9 clones yield another sequence named as LY-13, 2 clones yield a sequence named as LY-7/43, 2 clones yield a sequence named as LY-27/45, and the remaining 2 clones each yield one sequence named as LY-32 and LY-46. The dissociation constant for the selected aptamers were measured using flow cytometry, as shown in Table 1, aptamer LY-1 and LY-13 recognize the target cells with apparent Kds of 167.3 ± 30.2 nM and 185.6 ± 28.3 nM, respectively. Other aptamers possess Kds in high nanomolar ranges indicating low affinity for target cell (see Table 1).

**Table 1:** Sequences and Kds of selected aptamers for the metastatic HCCLM9 cells

Aptamers	Sequences*	Kd (nM)
**LY-1**	TTGGGTGTTAGGCTGGTCTTAATCGGGTCGGGTTGCTG	167.3 ± 30.2
**LY-13**	TAGCTGATTGGGTGGCGGGCATGTTCCGCTGGGGTCGGG	185.6 ± 28.3
**LY-32**	TGAGGTGGGTTTTCGGTCGGTTGTCTTGGTGTTTTGGTGC	245.7 ± 44.4
**LY-46**	TGAGGGGGGTTTTCGGTCGGTGTTATTGTTTTGTGTTTGC	262.5 ± 39.8
**LY-27/45**	CAGGGGTTAGGGTGCAGGGGGGGTCGTGTGGGGTCGTCTC	303.6 ± 34.5
**LY-7/43**	GCCGGGGCTGGGGTTGGGATGGCGGATGTGGGAGGGCTGA	369.7 ± 46.3

* the primer regions of aptamers are excluded

### Analysis of binding specificity of selected aptamer LY-1

**Figure 2:**
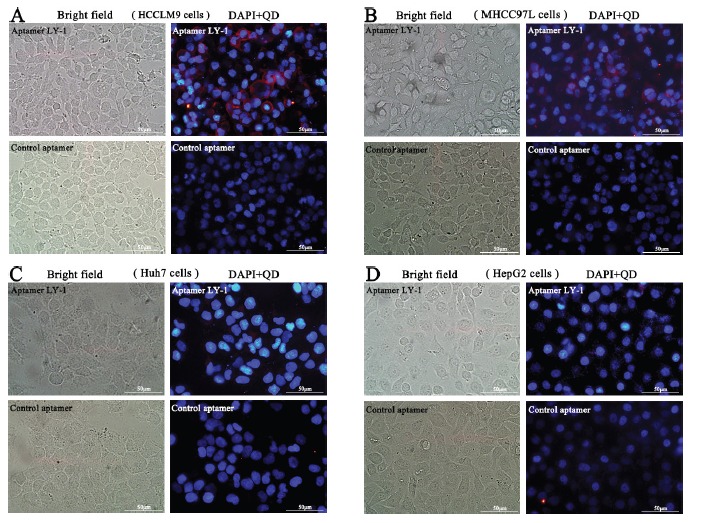
Fluorescent images showed that aptamer LY-1 specifically bound to the surface of HCCLM9 cells **A.** but not non-metastatic MHCC97L cells **B.** Huh7 cells **C.** or HepG2 cells **D.**

**Figure 3:**
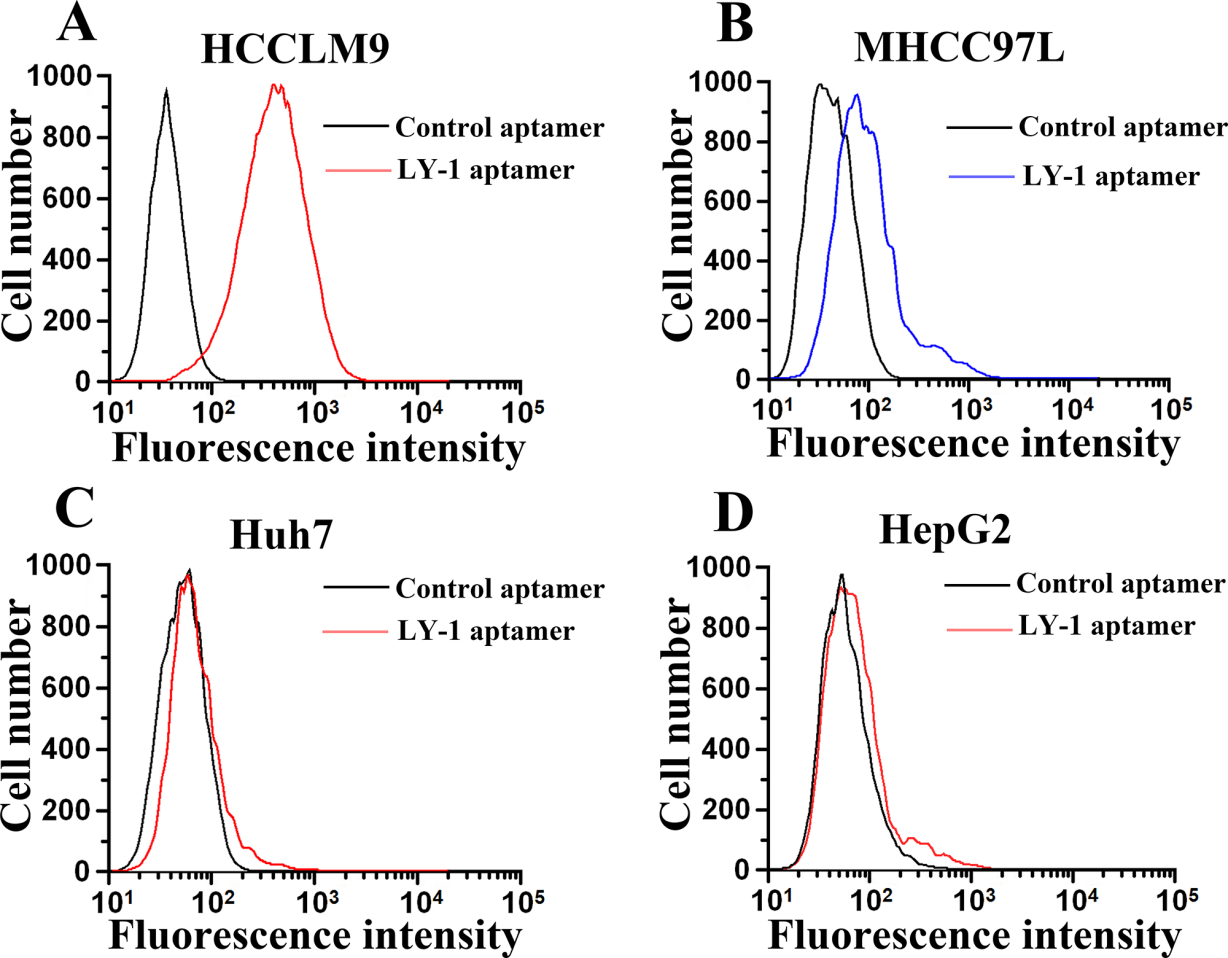
Flow cytometry analysis demonstrated that fluorescence labeled aptamer LY-1 can bind to HCCLM9 cells **A.** but not non-metastatic MHCC97L cells **B.** Huh7 cells **C.** or HepG2 cells **D.**

Based on the results of sequence analysis, apparent dissociation constants (Kd) and dominant position among all of identified aptamers, we selected LY-1 for further study. In order to determine the binding specificity of LY-1, HCCLM9 cells (Figure 2A and Figure 3A), MHCC97L cells (Figure 2B and Figure 3B), Huh7 cells (Figure 2C and Figure 3C) and HepG2 cells (Figure 2D and Figure 3D) were staining with QD605 and FITC labeled LY-1 and subjected to fluorescent imaging (Figure 2) and flow cytometry analysis (Figure 3) respectively. We found that LY-1 exhibited high specific binding to HCCLM9 and low or no binding to the rest of HCC cells, indicating LY-1 only recognizes targets present on the surface of metastatic HCC cells.

### Aptamer LY-1 target proteins on the surface of HCCLM9 cells

**Figure 4:**
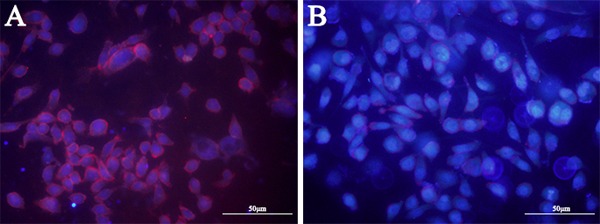
Treatment of proteinase K disrupts binding signal of aptamer LY-1 on the surface of HCC Pretreatment of proteinase K for 10 mins abolished binding sites of LY-1 on the surface of HCCLM9 cells **B.** as compared with untreated HCCLM9 cells **A.**

In order to investigate the category of possible binding molecules of LY-1, HCCLM9 cells were treated with proteinase K. Treatment of proteinase K has been shown to effectively digest extracellular domains of membrane protein without disrupting other plasma membrane components, such as lipids, sugars and other small organics. As shown in Figure 4, 10 min treatment of proteinase K completely abolished binding signal of QD605 labeled LY-1. Thus, these results clearly suggested that the targets of LY-1 are most likely membrane proteins.

### QD605 labeled aptamer LY-1 recognizes HCC cells in both local liver cancer tissues and pulmonary cancer metastatic tissues

**Figure 5:**
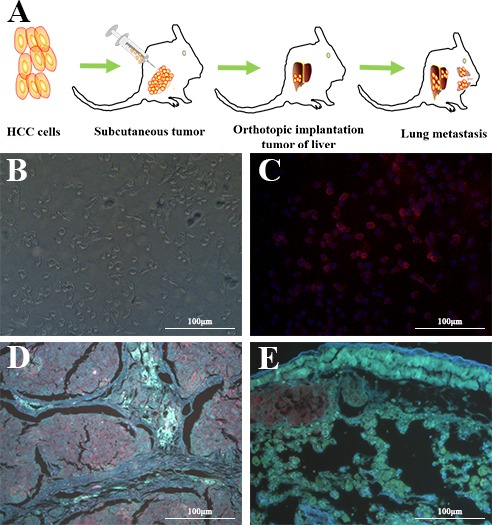
QD605- aptamer LY-1 labels local HCC cells in liver and metastatic HCC cells in lung tissues **A.** Nude mice model of HCC with spontaneous pulmonary metastasis, **B.** the HCCLM9 cells in bright field, **C.** The QD605 conjugated aptamer LY-1 fluorescence imaging of HCCLM9 cells, **D.** Representative fluorescent image shows aptamer LY-1 bound to the HCC cells in local mouse liver tumor tissues; **E.** aptamer LY-1 bound to the metastatic HCC cells in lung tissues.

To further explore whether LY-1 can serve as an *in vivo* biomarker to recognize HCC cells in both local liver tissues as well as remote metastatic sites, a xenograft model of HCC with pulmonary metastasis was employed in this study. As shown in Figure 5, both local liver cancer tissues (Figure 5D) and lung metastatic tissues (Figure 5E) can be recognized by QD605 labeled LY-1 aptamer. Thus, LY-1 can serve as a marker for detection of metastatic HCC cells.

### Aptamer LY-1 targets to metastatic HCC with enhanced expression of CK19 and Vimentin

**Figure 6:**
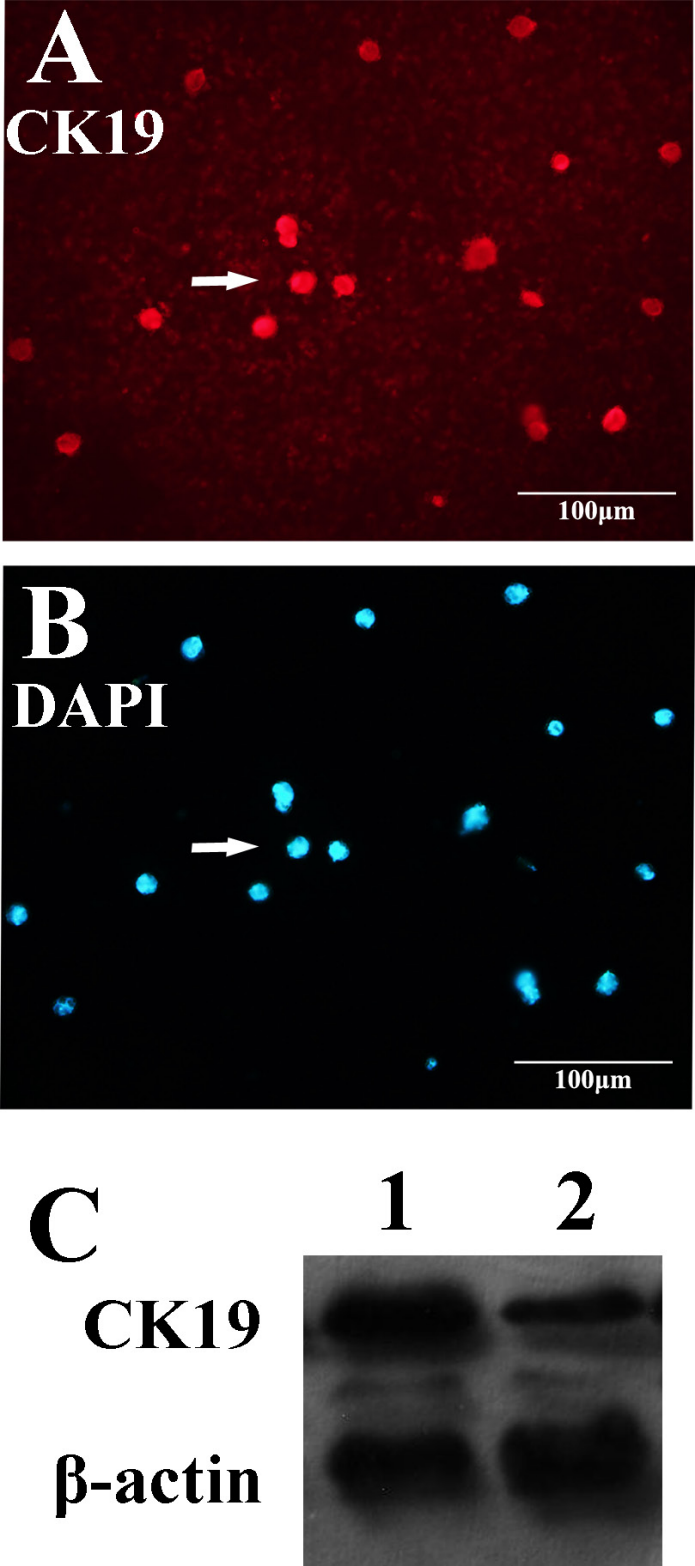
Aptamer LY-1 targets HCC that express more CK19 Western blot analysis showed that the cell lysate derived from aptamer LY-1 bound cells expressed more CK19 **C.** than that from aptamer LY-1 unbound cells. Immunostaining of CK19 **A.** confirmed the findings of Western blotting. DAPI was used to staining nucleus of cells **B.**

**Figure 7:**
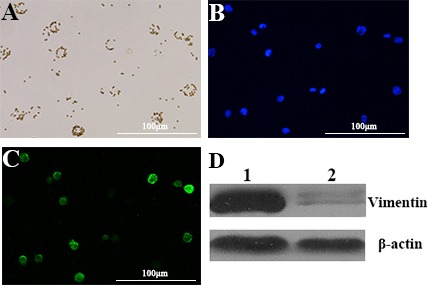
Aptamer LY-1 targets HCC that express more Vimentin Western blot analysis showed that the cell lysate derived from LY-1 bound cells expressed more Vimentin **D.** than that from LY-1 unbound cells. Immunostaining of Vimentin **C.** confirmed the findings of Western blotting. DAPI was used to staining nucleus of cells. **B.** The HCCLM-9 cells captured by magnetic-beads conjugated bio-aptamer LY-1 in bright field **A.**

Cancer cells are a population of uncontrolled growing cells with high level of genomic instability. During the progression of cancers, many factors within cancer cells will change to facilitate their proliferation and migration. In order to further dissect the molecular basis of targeting cells of LY-1, magnetic beads conjugated LY-1 was incubated with HCCLM9 cells. Then the cell lysates derived from LY-1 bound and unbound HCCLM9 cells were subjected to Western blotting analysis. As shown in Figure 6C and Figure 7D, LY-1 bound HCCLM9 cells expressed higher level of CK19 and Vimentin. These findings were further confirmed by immunostaining of LY-1 bound cells with either CK19 or Vimentin antibody (Figure 6A and Figure 7C). As several studies have demonstrated that overexpression of CK19 and Vimentin in HCC cells was positively correlated to metastatic behavior. These results further indicated that LY-1 has the capacity to bind to a sub-population of cells with higher migratory and invasive abilities.

### Aptamer LY-1 inhibits migration and invasion of HCCLM9 cells *in vitro*

**Figure 8:**
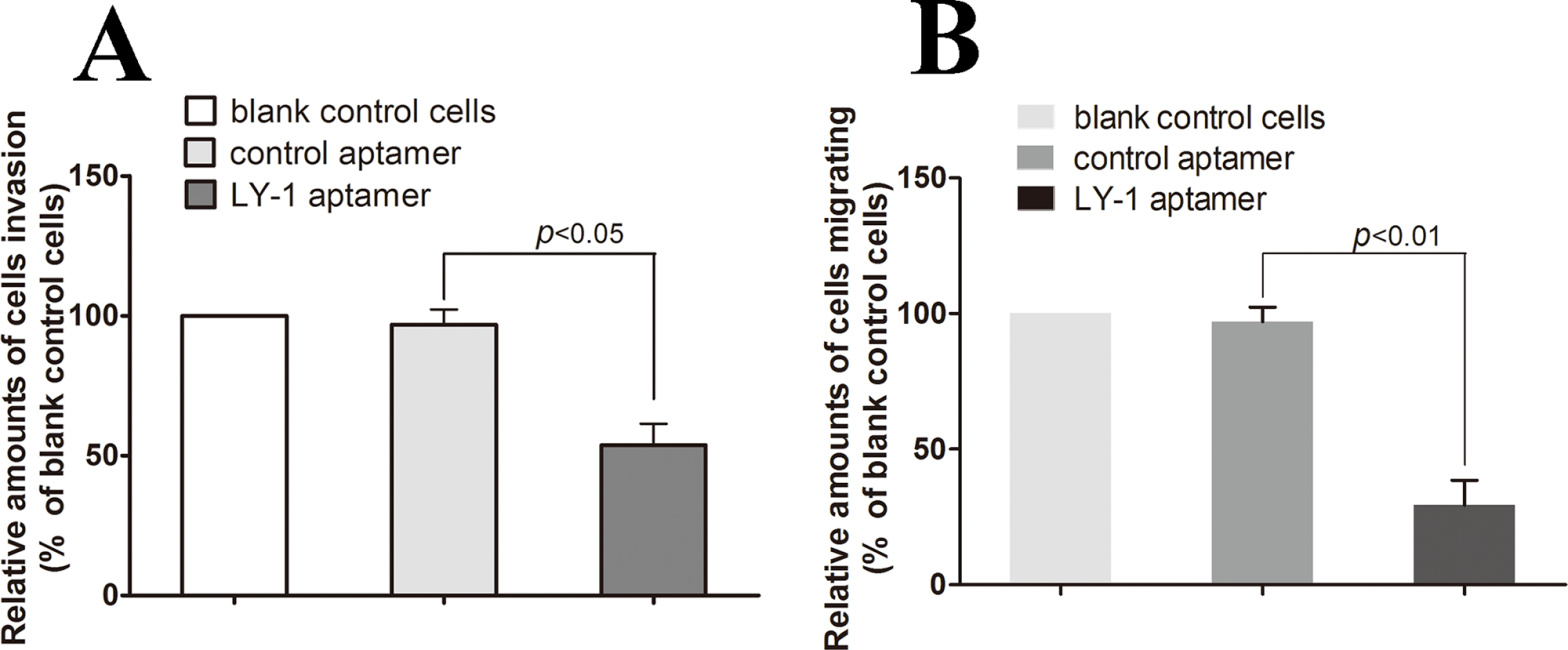
Aptamer LY-1 treatment inhibits migration and invasion of HCCLM9 cells *in vitro* To find out whether the aptamer LY-1 aptamer could inhibit metastatic HCC cells migration and invasion *in vitro*, the capacity of LY-1 aptamer-treated cells to penetrate noncoated **A.** and basal membrane-coated (Matrigel) **B.** porous membranes were analyzed. Invasion and migration of untreated cells (blank control cells) was used as a positive control (100%). Invasion and migration of cells treated with LY-I aptamer was represented as percentage of untreated control cells (% blank control cells). Values represent the mean of three independent experiments performed in triplicate. Statistical significance of the differences in migration and invasion of LY-1 aptamer-treated HCCLM9 cells refers to that of control aptamer-treated HCCLM9 cells. Data were presented as mean ± S.D.

Since we have shown that LY-1 can specifically bind to metastatic cells, our next question was whether binding of LY-1 could regulate behaviors of metastatic HCC. Thus, we analyzed the capacity of the cells to penetrate noncoated and basal membrane-coated (Matrigel) porous membranes, as quantitative indications of their migration and invasion abilities. As shown in Figure 8, treatment of LY-1 significantly inhibited cell migration (29.3 ± 3.06 % of LY-1 treated cells *vs* 97.1 ± 2.11 % of blank control cells, Figure 8A), and cell invasion (53.8 ± 4.36 % of LY-1 treated cells *vs* 96.83 ± 3.14 % of blank control cells, Figure 8B). Taken together, our data indicated that treatmentof LY-1 can inhibit both migration and invasion of the metastatic cells *in vitro*.

### Aptamer LY-1 treatment inhibits tumor growth in a HCCLM9 cell xenograft mouse model

**Figure 9:**
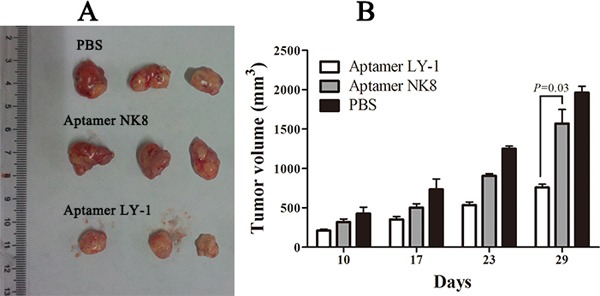
Aptamer LY-1 suppresses tumor growth in a xenograft model of HCC Tumor bearing nude mice was treated with PBS, control aptamer NK8 and LY-1 through I.P. injection. Tumor volume was recorded **B.** at indicated time points. At the end of experiments, xenografts were carefully dissected and photographed **A.** Data were presented as mean ± S.D. p = 0.03.

To further test the anti-tumor effect of LY-1 *in vivo*, we employed a xenograft mouse model of HCC. We injected the cells (2×10^6^) subcutaneously into the flanks of nude mice. Five days after the injection, the animals were divided into three groups and administered with PBS as control (group 1), or 0.25 mg/mice of negative control aptamer NK8 (group 2) or aptamer LY-1 (group 3) by intraperitoneal injection three times a week. Tumor volumes were evaluated at 10,17,23,29 days. 29 days after injection of the cells, the mice were sacrificed and the tumors were harvested and evaluated. As the mean tumor volume shown in Figure 9, there was no difference in the between the Groups injected with PBS (group 1) and negative control aptamer NK8 (group 2). In contrast, the animals treated with aptamer LY-1 (group 3) showed significantly reduced tumor growth. The tumor volume decreased 60% in the aptamer LY-1 injected group as compare with either PBS control or control aptamer NK8 treated group (tumor volume on 29 days after injection, 1962 ± 139.8 mm^3^ in PBS, 1569 ± 308.2 mm^3^ in negative control aptamer NK8 versus 757.4± 70.68 mm^3^ in aptamer LY-1, *p* = 0.03) (Figure 9B). In addition, the toxicity of all treatments was evaluated by evaluation of the behavioral changes of mice after treatment and monitoring the body weight of mice. The results showed that none of the mice displayed noticeable abnormal behavioral change, nor significant change in body weight, indicating the aptamers were not toxic (data not shown). Taken together, the data suggested that aptamer LY-1 can be potentially used for treatment of HCCs with minimal toxicity.

## DISCUSSION

Molecular medicine is an emerging field with focus on understanding the molecular basis of diseases and translating this information into strategies for diagnosis and therapy [22]. This approach could lead to personalized medical diagnosis and treatment. The ability to understand metastatic-HCC at the molecular level is limited due to the lack of molecular tools to identify and characterize the distinct molecule expression profiling [23, 24]. Currently, to address this problem, aptamers, which are single-stranded DNA or RNA oligonucleotides with high selectivity, affinity and stability, have drawn considerable attention for biomedical application [25]. These oligonucleotides, which are selected by an *in vitro* process known as cell-SELEX, have demonstrated a great potential in the study of molecular bases of cancer cells for cancer diagnosis and therapy [18, 26].

Recently, aptamer selection against live cells has been demonstrated and increasingly adopted. Using the cell-SELEX, aptamers have been selected for several cancer cell types, including acute lymphoblastic leukemia, small cell and non-small cell lung cancer, liver cancer (mouse), B cell lymphoma, acute myeloid leukemia, colorectal cancer and breast cancer [6, 18, 25, 27–29]. However, there is no study on aptamer specifically targeting metastatic HCC. In our study, we adopted the modified subtractive cell-SELEX method, using the high metastatic potential human HCC cell as target cell and the low metastatic potential human HCC cell as subtractive cell, through 9 round selections and identification by flow cytometry and fluorescence imaging, and acquired the high affinity and specific aptamer named as LY-1. To test whether the aptamer LY-1 could be used for HCCLM9 cell specific recognition, other HCC cells including MHCC97L and Huh7 and HepG2 were selected. The aptamer LY-1 showed specific response to the target cell and little or no response to other HCC cells. The aptamer LY-1, as a specific molecular probes, provide basis for clinical treatment of the metastasis of HCC.

Several studies have indicated that cytokeratins play an important role in the regulation of cell migration and invasion[30]. Cytokeratin 19 is the smallest member of cytokeratins (CKs) family, a group of heterogeneous intermediate filament proteins. CK19 is clinically used as a discrimination marker of HCC and intrahepatic cholangiocarcinoma[31]. However, some HCC cells and tumor again express CK19 during tumorigenesis. Wu et al[32] showed that some HCC cell lines did express CK19, and the CK19 positive HCC cells tended to be poorly differentiated and more aggressive. Ding et al [30] demonstrated that CK19 was overexpressed in high metastatic MHCC97H cells as compared with low metastatic MHCC97L cells. Moreover, CK19 expression is gradually increased in line with the metastatic potency of HCC cell with progressively increasing spontaneous metastatic potentials, implying the possible role of CK19 in metastasis.

To identify the molecular identity of metastatic HCC cells that can be recognized by LY-1, we separated the subpopulation of metastatic HCC cells using the aptamer LY-1 conjugated magnetic beads. Through the immunofluorescent and Western blot analysis, we found that CK19 and Vimentin expression was higher in the captured metastatic HCC cells, implying the subpopulation of HCC cells with more invasive and migration potential. However, we cannot address every aspect of LY-1 related question in the current study, for example, we still don't know the specific target(s) of LY-1 on the surface of metastatic HCC cells. It will be the future direction of our research. Although we don't have evidence to demonstrate the target(s), there are several candidates, for example, previous study has shown that coronin-1C overexpresses in MHCC97H cells as compared with their low metastatic counterpart [19], we also showed that CK19 overexpressing cells can be targeted by LY-1, thus, it is possible that coronin-1C or CK19 could be the potential targets for LY-1. We also plan to utilize proteomic approach to identify more potential targets (please see Supplementary Materials and Supplementary Figure S2 and Supplementary Table S1 for more preliminary findings).

In addition to specific recognition abilities, some aptamers can directly interrupt a disease process or trigger their therapeutic effects through binding disease-related proteins on the cell surface, and further modulate the biological activities of the molecular targets involved in pathogenesis [5, 22, 33]. Since cell-binding aptamers possess excellent targeting properties, they hold a great potential for targeted cancer therapy as therapeutic agents.

Currently, a number of studies have focused on the therapeutic application of aptamers derived by cell-SELEX [10]. Cerchia et al [34] used intact PC12 cells as the target to select RNA aptamers. The preferred aptamers D4 and D24 could hinder RET downstream signaling and subsequent molecular and cellular events. Zueva et al. [5] used two malignant isogenic hamster cell lines HET-SR-1(for positive selection) and HET-SR (for counter-selection) to select DNA aptamers. The preferred aptamers E10 and E37 disrupted migration and invasion of tumor cells by decreasing the phosphorylation of multiple metastasis-associated tyrosine kinases. In another study, Cerchia's group [35] generated aptamers able to inhibit a specific intracellular signal transduction pathway and showed functional activity against tumor cell proliferation.

Metastatic recurrence is the most important biological behavior of HCC and the main cause of treatment failure [1, 19]. Thus, the inhibition of invasion and migration is of great importance for anti-HCC therapy strategies. Here, we hypothesized that subtractive cell-SELEX focusing on metastatic HCC cell phenotype could generate aptamers able to disrupt key aspects of particular importance for HCC cell migration and invasion. In our study, we found that the aptamer LY-1 inhibited HCC cells migration with a statistically significant 3.3-fold decrease and inhibited cell invasion with a 1.8-fold suppression (with respect to control aptamer). These results presented here suggest that aptamer LY-1 inhibiting the metastatic spread by targeting cell migration and invasion is considered as promising agent for HCC therapeutic purposes.

In summary, we developed a SELEX strategy for identifying aptamers that could distinguish molecular targets expressed by two strictly isogenic and equally tumorigenic cancer cell lines, phenotypically differing only by their *in vivo* metastatic potential. It is showed that this molecular evolution approach effectively allows obtaining aptamer LY-1 recognizing the metastatic HCC cell with high affinity and specificity. Most interestingly, the aptamer LY-1 could inhibit invasion and migration of HCC cells grown *in vitro*. *In vivo* study also indicated LY-1 can label both HCC cells in liver tissues and lung metastasis tissues. The present study provides evidence indicating the aptamer LY-1 is a promising therapeutic agent in metastasis of HCC. Data from our present study implicate a promising HCC therapy that blocks HCC metastasis by aptamer LY-1 and a promising molecular typing probe for prognosis in metastasis of HCC.

## MATERIALS AND METHODS

### Antibodies and reagents

Rabbit anti-human cytokeratin (CK) 19 monoclonal antibody was purchased from R&D Systems. Yeast tRNA, BSA and Salmon sperm DNA were purchased from Fisher Scientific (Thermo Fisher Scientific Inc., USA). Streptavidin coated magnetic beads (Dynabeads, M-280 Streptavidin) used for separating the single strand DNA and capturing cells were purchased from Invitrogen. The AmpliTaq Gold 360 PCR Master Mix was purchased from ABI (Applied Biosystems Inc., Foster City, CA). The cell-SELEX DNA library contained a 40-base central random sequence flanked by primer sites on either side (5′-ATC CAG AGT GAC GCA GCA-N40-TGG ACA CGG TGG CTT AGT-3′). The FITC-labeled forward primer (5′-FITC-ATC CAG AGT GAC GCA GCA-3′) and biotin-labeled reverse primer (5′- Bio-ACT AAG CCA CCG TGTCCA-3′) were used in PCR to get the double-labeled DNA and to separate the single stranded DNA by streptavidin-coated magnetic beads. The FITC-labeled sequences were used to monitor progress of selection by flow cytometry (Beckman Coulter, USA). The control aptamer was NK8 bound to Mycobacterium tuberculosis[20]. All sequences were synthesized by SBS Genetech Co., Ltd (Shanghai, China) and purified by reverse phase high performance liquid chromatography (HPLC) (Agilent Technologies, USA).

### Cell lines and cell culture

Low metastatic MHCC97L cells and high metastatic HCCLM9 cells were established from parental cell line MHCC97. Other HCC cell lines HepG2 and Huh7 were maintained at our laboratory. These cells were cultured in high glucose DMEM with 10% FBS and incubated at 37°C in a humidified atmosphere containing 5% CO_2_.

### *In vitro* selection of HCC metastasis-related aptamers using subtractive cell-SELEX

*In vitro* selection was carried out as described [7] with a number of modifications. The procedures of selection were as follows. HCCLM9 cell was used as target cell and MHCC97L for subtractive selection as control cell. The ssDNA library (8 nmol) was first denatured at 95°C for 5 min and kept on ice for 10 min, then dissolved in 1 mL pre-cooling binding buffer (PBS-1M MgCl_2_-0.1 mg/mL yeast tRNA-1 mg/mL BSA-0.1 mg/mL Salmon sperm DNA). Target cells (1×10^7^) were washed, dissociated (PBS −0.02% EDTA), and then incubated with the ssDNA library on ice in an orbital shaker for 60min. After incubation, the cells were washed three times to remove unbound DNA sequences. Add 500μl of DNase-free water to the adhesive cells and scraped off, resuspend cells and transfer cell suspension into a 1.5-mL microfuge tube, heat the cell mixture at 100°C for 5 min, centrifuge at 13,100 g for 5 min and collect supernatant containing eluted ssDNA. The bound sequences were amplified by PCR using FITC- and biotin-labeled primers. Amplifications were carried out in ABI 9600 Cycler (Applied Biosystems Inc., Foster City, CA, USA) at 95°C for 40 sec, 56°C for 40 sec, and 72°C for 40 sec, followed by the final extension for 7 min at 72°C. The selected sense ssDNA strands were separated from the biotinylated antisense ssDNA by streptavidin-coated magnetic beads and alkaline denaturation. For the fourth round of selection, the selected DNA pool could be used to carry out subtractive selection to filter out sequences that may bind to the molecules existing on the surface of both the target and control cell lines. To evolve the aptamers with high affinity and specificity, the wash strength was enhanced gradually by extending the wash time and increasing the volume of wash buffer and the number of washes, Furthermore, the target cell number and the concentration of ssDNA pool and the incubation time were gradually reduced. The entire selection process was repeated according to the extent of enrichment. The SELEX progress was monitored by flow cytometry. After 9 rounds of selection, the selected ssDNA pool was PCR-amplified using unmodified primers and cloned into *Escherichia coli* using the TA cloning kit (Invitrogen). Cloned sequences were determined by Invitrogen Co. Ltd (Shanghai, China).

### Flow cytometry analysis

To monitor the binding of enriched ssDNA pool during cell-SELEX, FITC-labeled ssDNA pools were incubated with target cells HCCLM9 or subtractive cells MHCC97L (1×10^6^ ) in 500 μL binding buffer on ice for 30 min. Cells were washed twice after incubation and the fluorescence intensity was determined by flow cytometry. The FITC-labeled control aptamer NK8 (bound to Mycobacterium tuberculosis) was used as a negative control. The 3, 5, 7, and 9 rounds ofenriched ssDNA pool were analyzed by flow cytometry (Beckman Coulter, USA).

To determine the binding affinity constant of the selected aptamers, target cells (1×10^6^) were incubated with varying concentrations of FITC-labeled aptamers in 500 μL binding buffer on ice for 30 min. Cells were washed twice after incubation and the fluorescence intensity was determined by flow cytometry. The FITC-labeled control aptamer NK8 was used as a negative control. All binding assays were in triplicate. The mean fluorescence intensity of the control aptamer NK8 was subtracted from that of the selected aptamer with the target cells to determine the specific binding. The equilibrium dissociation constant (Kd) of the aptamer-cell interaction was obtained by fitting the dependence of intensity of specific binding on the concentration of the aptamers to the equation Y = B max X/(Kd + X) (B max and X), using Prism software (V5.0, GraphPad Software, Inc, CA, USA).

To determine the cell specificity of the selected aptamer, human cancer cell lines including HCC cell lines MHCC97L, HepG2 and Huh7 were used in binding assays by flow cytometry.

### Fluorescence microscopy imaging of cells stained with aptamer

Cells were cultured in chamber slides, grown overnight, and rinsed with PBS. For fluorescence imaging, the selected individual aptamer labeled with FITC or quantum dot -QD605 was incubated with cell monolayer in chamber slides in binding buffer on ice for 30 min. After washing, cells were imaged with Olympus BX51 fluorescence microscope equipped with an Olympus DP72 camera (Olympus Optical Co., Ltd., Tokyo, Japan).

### *In vitro* cell invasion and migration assays

Cell invasion and migration were assayed according to the methods described by Zueva E et al [5]. Cells were serum-starved in the presence of the aptamer LY-1 or the control aptmer NK8 for 24h, seeded to Boyden chamber (Neuro Probe, Cabin John, MD, USA) at 1×10^5^ cells/well in serum free medium and then incubated for 24h at 37°C. For invasion assay, 10 μL Matrigel (25 mg/50 mL; BD Biosciences, MA, USA) was applied to 8 μm pore size polycarbonate membrane filters and the bottom chamber contained standard medium. Cells were allowed to invade through the membrane for 24h in serum-free growth medium in the presence of the aptamer LY-1 or the control aptmer NK8, toward the lower chamber containing growth medium supplemented with 10% fetal bovine serum and the aptamer LY-1 or the control aptmer NK8. The invaded cells were fixed with 100% methanol and stained with 0.1% crystal violet, and extensively washed with PBS. For quantification, cell numbers were counted in four independent areas of the membrane under a light microscope at ×200 magnification. Three independent experiments were performed, each in triplicate. The migration assay was carried out as described in the invasion assay with no coating of Matrigel.

### Nude mice model of HCC spontaneous pulmonary metastasis

Male athymic BALB/c nu/nu mice, 4-wk old, were obtained from Beijing HFK Bio-Technology Co., Ltd and maintained in an Animal Biosafety Level 3 Laboratory at the Animal Experimental Center of Wuhan University. The protocols were approved by the Animal Care Committee of Wuhan University. HCCLM9- nude mice were produced as described previously [21]. All mice were sacrificed under deep anesthesia by peritoneal injection of 3% pentobarbital sodium in approximately 6 wks after model construction. Liver and lung samples were collected and stored at −80°C refrigerator. Some lung samples were fixed in 10% neutral formalin solution and embedded in paraffin. Four micrometer thick sections were cut from each paraffin block and stained with aptamer LY-1 labeled with quantum dot (QD605) to visualize local liver tumor tissues and lung metastasis.

### Immunofluorescence imaging analysis

Target cells (1×10^5^) were washed, dissociated (PBS-0.02% EDTA), and then incubated with the biotin-labeled aptamer LY-1 on ice in an orbital shaker for 30 min. After incubation, the cells were washed 3 times to remove unbound DNA sequences. After washing, the cells were then incubated with streptavidin-coated magnetic beads (Dynabeads, M-280 Streptavidin, Invitrogen) at room temperature for 30 min. After washing 3 times in a magnet, the suspension of the cells-aptamer-magnetic beads was dropped on the glass slides pre-treated with paraformaldehyde. After dry, the glass slides were washed in PBS and fixed in methanol at −20°C for 5 min. The cells were first incubated with anti-human keratin 19 primary antibody overnight at 4°C, washed and then incubated with PE-conjugated anti-rabbit IgG secondary antibody (dilution 1:200, Santa Cruz Biotechnology Inc) for 60 min at room temperature. After washing, stained cells were imaged with Olympus BX51 fluorescence microscope equipped with an Olympus DP72 camera.

### Western blot analysis

Target cells (1×10^6^) were washed, dissociated (PBS-0.02% EDTA), and then incubated with the biotin-labeled aptamer LY-1 on ice in an orbital shaker for 30 min. After incubation, the cells were washed 3 times to remove unbound DNA sequences. After washing, the cells were then incubated with streptavidin-coated magnetic beads (Dynabeads, M-280 Streptavidin, invitrogen) at room temperature for 30 min. After washing 3 times in a magnet, the suspension of the cells-aptamer-magnetic beads were boiled in 0.1% SDS for 5 min, and the suspension for western blot analysis was collected. The HCC cell lysates (HCC cells captured by aptamer-conjugated magnetic beads and the remaining HCC cells after capture) were separated in a 10% polyacrylamide gel and transferred onto a nitrocellulose membrane. The blot was subsequently incubated with 5% non-fat milk in Tris-buffered saline (20 mM Tris, 137 mM NaCl, pH 7.6) for 60 min to block non-specific binding and then overnight with antibody against CK19. Blots were then incubated with a horseradish peroxidase-conjugated goat anti-rabbit IgG (dilution 1:1000, Santa Cruz Biotechnology Inc) for 60 min. After washing, signal was detected by using enhanced chemiluminescence (ECL) commercial kit (Amersham Biosciences).

### Protease treatment for HCCLM-9 cells

The cultured HCCLM9 cells were washed twice with PBS, then incubated with 1 mL Trypsin-free cell dissociation solution (PBS-0.02% EDTA) or 1 mL Trypsin-free cell dissociation solution (PBS-0.02% EDTA) containing 0.1 mg/mL proteinase K in PBS at room temperature for 10 min. Pre-cooled PBS (containing 10% FBS) was then added to quench the proteinase. Then the treated cells (1×10^6^) were subjected to quantum dot -QD605 labeled LY-1 for fluorescent imaging by Olympus BX51 fluorescence microscope equipped with an Olympus DP72 camera.

### Xenograft model of HCCLM9 cells in nude mice

5-week-old BALB/c-nude mice were purchased from Beijing HFK Bio-Technology Co., Ltd and maintained in an Animal Biosafety Level 3 Laboratory at the Animal Experimental Center of Wuhan University. The protocols were approved by the Animal Care Committee of Wuhan University. The animals were kept under specific pathogen-free conditions and acclimated to laboratory conditions for at least 1 week before use. The xenograft model of HCC was established by subcutaneous injection of 2×10^6^ HCCLM9 cells into the flanks of mice. Five days after inoculation, the mice were randomly divided into three groups (*n* = 3 per group): **(1)** PBS buffer control, **(2)** Neagative control aptamer NK8, **(3)** aptamer LY-1, and administered with aptamer (10 mg/kg) in 50ul of PBS by intraperitoneal injection three times a week for 29 days. Tumor volumes were evaluated at 10,17,23,29 days. Finally, the mice were sacrificed and the tumors were harvested and evaluated.

### Statistical analysis

The software of SPSS version 13.0 for Windows (SPSS Inc, IL, USA) was used for statistical analysis. Statistical significances in this study were analyzed by Student's t-test to compare differences between treatments. A difference at p < 0.05 was considered to be statistically significant and the experiments were repeated three times.

## SUPPLEMENTARY DATA FIGURES AND TABLE


