# Supplementary material for: Identification of an aptamer through whole cell-SELEX for targeting high metastatic liver cancers
Source: Oncotarget. 2016 Jan 23;7(7):8282–94. doi: 10.18632/oncotarget.6988 (PMC4884992; doi:10.18632/oncotarget.6988)
Supplement: Supplementary file 1 [file oncotarget-07-8282-s001.pdf]

SUPPLEMENTARY DATA

IDENTIFICATION OF TARGET PROTEIN BOUND TO APTAMER LY-1

METHOD

Aptamer LY-1 target pull-down and identification of aptamer-bound protein

HCCLM9 cells (1 × 10^8 cells) were washed twice with pre-cooled hypotonic buffer [50 mmol/L Tris-HCl (pH 7.5)] and then incubated with the same hypotonic buffer containing protease inhibitors at 4°C for 30 minutes. The buffer was completely removed, and after 2 washes, the cells were lysed in 0.5 mL of membrane lysis buffer (PBS containing 5 mmol/L MgCl2 and 1% Triton X-100) containing protease inhibitors at 4°C for 30 minutes. After centrifugationat 14000 g for 15 min, the supernatant were separated in a 10% polyacrylamide gel and transferred onto a nitrocellulose membrane. The blot was subsequently incubated with 5% non-fat milk in Tris-buffered saline (20 mM Tris, 137 mM NaCl, pH 7.6) for 60 min to block non-specific binding and then overnight with biotinylated Aptamer LY-1. Blots were then incubated with a horseradish peroxidase-conjugated Streptavidin (dilution 1:2000, Santa Cruz Biotechnology Inc) for 60 min. After washing, signal was detected by using enhanced chemiluminescence (ECL) commercial kit (Amersham Biosciences).

The supernatant also was incubated with 500 pmol of aptamer LY-1-biotin or biotinylated aptamer NK8 at 4°C for 60 minutes along with a 100-fold excess concentration of yeast tRNA. The aptamer-protein complex was captured by further incubating with 1 mg (100 μl) of magnetic streptavidin beads at room temperature for 30 min.The beads with bound aptamer-protein complexes were then collected on an EasySep magnet stand and washed five times with 1 mL of the lysis buffer. The enriched proteins were heated for elution and separated by 10% SDS-polyacrylamide gel electrophoresis (SDS-PAGE). The gels were then silver-stained with the PierceSilver Stain Kit. The aptamer-specific protein bands were excised and trypsin-digested in situ and analysed by ESI-MS/MS that was conducted on a capillary system equipped with the Aksigent autosapmler (NanoLC-2Dsystem, US.).

RESULT

Identification of aptamer LY-1 targets via aptamer-mediated protein pull-down assay

The complete loss in Aptamer LY-1 binding to protease K treated HCCLM-9 cells confirmed that the target is a membrane protein. In addition, only partial loss in binding to enzyme-treated cells indicates that either the target protein has some resistance toward enzyme or is present in membrane domains that are inaccessible to enzyme. Identification of the target protein was conducted the aptamer-based western blotting analysis. As shown in Figure S2A, compared with the control aptamer NK8 bound protein band, we found that a distinct band around 42–35 kDa that was present in the aptamer LY-1 binding but not in the neagative control. This indicated that the band at around 42–35 kDa was specific to Aptamer LY-1.

Further identification of the target protein was conducted via biotinylated aptamer LY-1 and streptavidin-magnetic beads-based pull-down from the membrane lysate. Aptamer-bound proteins were eluted by RNAse I treatment and finally resolved on a precast gradient gel under denaturing conditions. As shown in Figure S2B, silver-stain (Because of low abundance protein, Colloidal Coomassie staining could not be used) revealed a faint band around 42–35 kDa that was present in the aptamer LY-1 pull-down. In addition, the absence of this band in the presence of high concentrations of biotin-free aptamer LY-1 competitor confirms that this is the target protein. Mass spectroscopic analysis of these bands, unforturenitly, not revealed a common match of membrane protein, most likely cytoskeletal proteins (Supplementary Table S1). The aptamer LY-1 target cell surface proteins were not further validated.

SUPPLEMENTARY FIGURES AND TABLE

Schematics of nude mice model of HCC with spontaneous pulmonary metastasis.

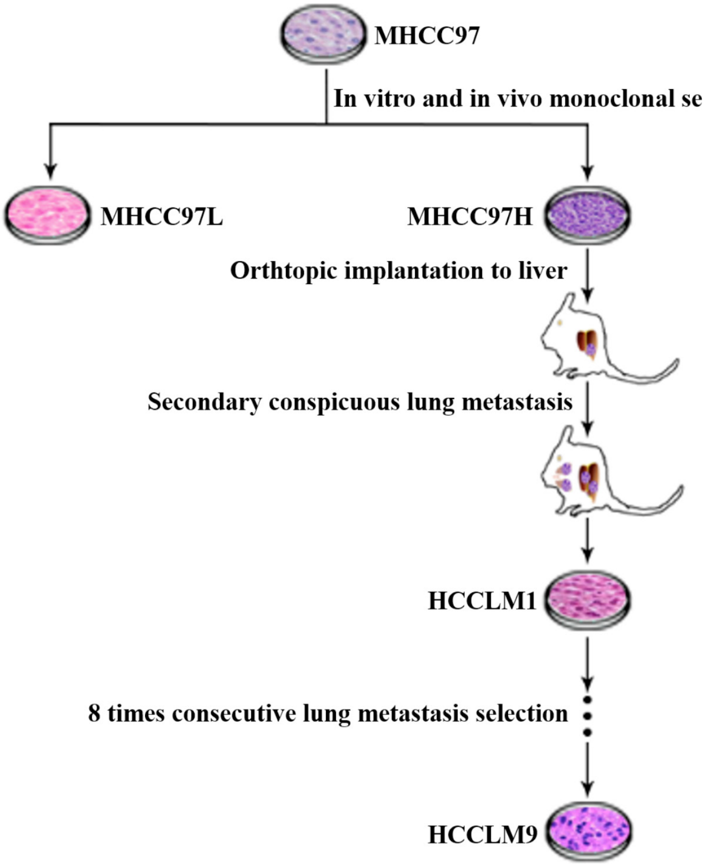

Supplementary Figure S1: Putative model of MHCC97L (low metastatic potential) and HCCLM9 (high metastatic potential) construction.

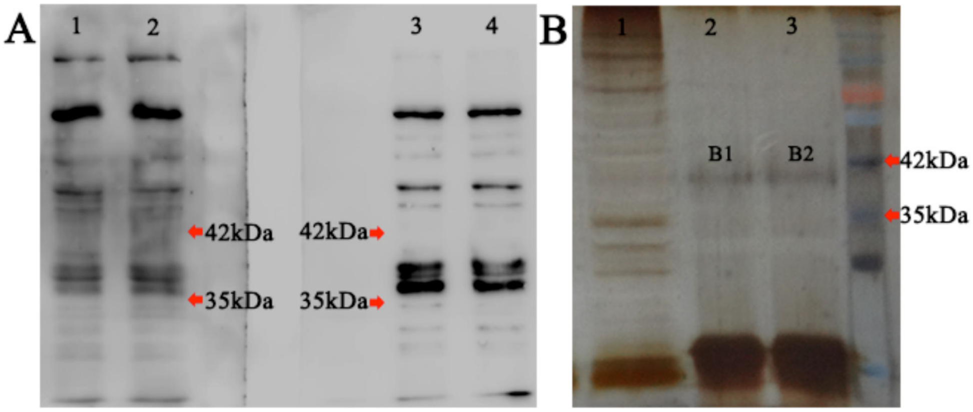

Supplementary Figure S2: Identification of aptamer LY-1 targets via aptamer-mediated protein pull-down assay. A. Identification of the target protein was conducted the aptamer-based western blotting analysis. Lane 1 and lane 2 are duplications, the control aptamer NK8 bound protein band; Lane 3 and lane 4 are duplications, the control aptamer LY-1 bound protein band. **B.** Further identification of the target protein was conducted via biotinylated aptamer LY-1 and streptavidin-magnetic beads-based pull-down from the membrane lysate. Silver-stain (B1, B2) revealed a faint band around 42–35 kDa that was present in the aptamer LY-1 pull-down. Lane 1, total lysis cell membrane protein; lane 2 and lane 3 are duplications, aptamer LY-1 pull-down proteins.

Supplementary Table S1: Mass-spectroscopy analysis data of aptamer LY-1specific bands (B1 and B2)

Protein Name	Accession	Summary Score	Peptides (95%)
Keratin, type II cytoskeletal 1	spP04264	56.76	26
Keratin, type II cytoskeletal 2 epidermal	spP35908	28.84	15
Keratin, type I cytoskeletal 9	spP35527	25.46	12
Keratin, type I cytoskeletal 10	spP13645	16.86	9
Keratin, type I cytoskeletal 14	spP02533	7.91	5
Hornerin	spQ86YZ3	6.87	4
Keratin, type II cytoskeletal 5	spP13647	4.46	4
Filaggrin-2	spQ5D862	3.89	2
Actin, cytoplasmic 2	spP63261	3.51	2
Actin, cytoplasmic 1	spP60709	0	2
Actin, alpha skeletal muscle	trQ5T8M8	0	2
Actin, alpha skeletal muscle	trQ5T8M7	0	2
Actin, cytoplasmic 2, N-terminally processed (Fragment)	trI3L4N8	0	2
Actin, cytoplasmic 2, N-terminally processed (Fragment)	trI3L3I0	0	2
Actin, cytoplasmic 2, N-terminally processed (Fragment)	trI3L1U9	0	2
Actin, alpha skeletal muscle	trA6NL76	0	2
Actin, alpha skeletal muscle	spP68133	0	2
Actin, alpha cardiac muscle 1	spP68032	0	2
Actin, gamma-enteric smooth muscle	spP63267	0	2
Actin, aortic smooth muscle	spP62736	0	2
Actin, cytoplasmic 2, N-terminally processed (Fragment)	trK7EM38	0	2
Actin, cytoplasmic 2, N-terminally processed	trJ3KT65	0	2
Actin, cytoplasmic 2, N-terminally processed (Fragment)	trI3L3R2	0	2
Actin, cytoplasmic 2	trG5E9R0	0	2
Actin, cytoplasmic 2 (Fragment)	trE7EVS6	0	2
Actin, gamma-enteric smooth muscle	trF8WB63	0	2
Actin, aortic smooth muscle (Fragment)	trF6UVQ4	0	2
Actin, aortic smooth muscle (Fragment)	trF6QUT6	0	2
Actin, cytoplasmic 2 (Fragment)	trC9JZR7	0	2
Actin, cytoplasmic 2 (Fragment)	trC9JUM1	0	2
Actin, cytoplasmic 2 (Fragment)	trC9JTX5	0	2
Actin, gamma-enteric smooth muscle (Fragment)	trC9JFL5	0	2
Actin, gamma-enteric smooth muscle	trB8ZZJ2	0	2
POTE ankyrin domain family member E	spQ6S8J3	0	1
POTE ankyrin domain family member I	spP0CG38	0	1

(*Continued*)

Protein Name	Accession	Summary Score	Peptides (95%)
POTE ankyrin domain family member F	spA5A3E0	0	1
POTE ankyrin domain family member J	spP0CG39	0	1
Actin, gamma-enteric smooth muscle	trF8WCH0	0	1
Prolactin-inducible protein	spP12273	1.74	1
Fructose-bisphosphate aldolase A	spP04075	0	2
